# Transcriptional-regulatory convergence across functional MDD risk variants identified by massively parallel reporter assays

**DOI:** 10.1038/s41398-021-01493-6

**Published:** 2021-07-22

**Authors:** Bernard Mulvey, Joseph D. Dougherty

**Affiliations:** Departments of Genetics and Psychiatry, Washington University in St. Louis, St. Louis, MO USA

**Keywords:** Genomics, Depression, Epigenetics in the nervous system

## Abstract

Family and population studies indicate clear heritability of major depressive disorder (MDD), though its underlying biology remains unclear. The majority of single-nucleotide polymorphism (SNP) linkage blocks associated with MDD by genome-wide association studies (GWASes) are believed to alter transcriptional regulators (e.g., enhancers, promoters) based on enrichment of marks correlated with these functions. A key to understanding MDD pathophysiology will be elucidation of which SNPs are functional and how such functional variants biologically converge to elicit the disease. Furthermore, retinoids can elicit MDD in patients and promote depressive-like behaviors in rodent models, acting via a regulatory system of retinoid receptor transcription factors (TFs). We therefore sought to simultaneously identify functional genetic variants and assess retinoid pathway regulation of MDD risk loci. Using Massively Parallel Reporter Assays (MPRAs), we functionally screened over 1000 SNPs prioritized from 39 neuropsychiatric trait/disease GWAS loci, selecting SNPs based on overlap with predicted regulatory features—including expression quantitative trait loci (eQTL) and histone marks—from human brains and cell cultures. We identified >100 SNPs with allelic effects on expression in a retinoid-responsive model system. Functional SNPs were enriched for binding sequences of retinoic acid-receptive transcription factors (TFs), with additional allelic differences unmasked by treatment with all-*trans* retinoic acid (ATRA). Finally, motifs overrepresented across functional SNPs corresponded to TFs highly specific to serotonergic neurons, suggesting an in vivo site of action. Our application of MPRAs to screen MDD-associated SNPs suggests a shared transcriptional-regulatory program across loci, a component of which is unmasked by retinoids.

## Introduction

Major depressive disorder (MDD) is a common but debilitating psychiatric disorder affecting hundreds of millions worldwide [1], exacting substantial tolls on both individuals [2] and societies [3]. Despite the global burden of MDD, nearly half of patients do not clinically respond to treatment [4], in part due to limited understanding of its biological underpinnings. Family studies have demonstrated that MDD risk is at least 30% heritable [5, 6]. More recently, genome-wide association studies (GWASes) have demonstrated similar estimates for severe MDD [7], and have helped narrow in on associated single-nucleotide polymorphisms (SNPs) [8–12], a tantalizing entry point for understanding the biology of MDD. However, GWAS studies do not identify causal variants, but rather implicate wider co-inherited regions consisting of many SNPs in linkage disequilibrium (LD). Most disease-associated SNPs are found outside of protein-coding sequences, suggesting probable roles in transcriptional regulation (TR) [13–16]. Which linked SNPs have functional allelic impacts on TR—and how they act together across loci to result in disease—remains unresolved.

It is thought that undetected, small-effect SNPs acting across the genome—including conditional SNPs within GWAS-significant loci [17]—contribute to the substantial heritability *not* caught by GWAS-significant SNPs alone [18]. Early support for multiple linked variants underlying GWAS signals came from examination of cell line histone marks in loci from six autoimmune disorder GWASes; all six showed enrichment of TR-suggestive marks at LD SNPs only in a pertinent cell type (B lymphocytes). Strikingly, 65% of the loci with ≥1 SNP overlapping lymphocyte histone marks contained multiple SNP-mark pairs, and over half of these loci contained at least three such SNPs [19]. Altogether, these findings implied that GWAS regions likely affect several TR features via several linked variants, especially in relevant cell types. More recently, GWASes have identified what are now called “conditional SNPs” associated with MDD [20]. However, despite predictions of multiple TR SNPs within GWAS loci, functional demonstration of this phenomenon has been sparse to date. The largest functional TR assay of MDD-associated variants examined 34 SNPs using luciferase assays [21], representing successful but low-throughput identification of functional MDD SNPs. However, in terms of broad linkage, these loci constitute well over 10,000 SNPs, which will ultimately require higher-throughput approaches.

Furthermore, how functional SNPs—even once identified—biologically result in disease remains unclear, given their individually small effects on risk. The polygenic [22] and omnigenic [18] models were conceived of to address these aspects of complex disease genetics, establishing a guiding principle for GWAS interpretation. In brief, these theories posit that consistent emergence of a specific phenotype via widespread genomic variation necessarily requires common biological endpoints of those variants’ effects. At the molecular level, these points of convergence could be either upstream (shared regulation across loci) [23] or downstream (common biological pathways across loci). For downstream analyses, myriad approaches have been developed to nominate gene targets of putative TR SNPs using proximity [24], chromatin structure [25–27], or expression quantitative trait loci (eQTLs) [28–30], yielding gene sets tested for enrichment in biological pathways [28, 29, 31] and cell types [32]. However, no analogous approaches exist for identifying convergent upstream (i.e., TR) molecular effects of genetic risk, in part because a prerequisite is defining the functional SNPs.

One possible point of upstream TR convergence of MDD risk variants is retinoic acid and related compounds (retinoids). Retinoids drive transcriptional responses via several retinoid-binding nuclear receptor transcription factors (TFs) and heterodimerizing partners [33, 34]. Besides their critical role in neurodevelopment, including of depression-implicated limbic structures [35], retinoids have been associated with MDD onset and suicidality by epidemiological studies of the retinoid agonist isotretinoin [36]. Moreover, thyroid hormone is often used as an adjunctive treatment in MDD, and thyroid receptor TR effects are frequently carried out cooperatively with RXR family retinoid receptors [37]. Additional evidence for retinoid pathway activity in the adult brain—and its overactivity as a risk factor for depression—comes from rodent pharmacology and genetic models. For example, knockdown of *Cyp26b1*—which metabolizes retinoids*—*in adult mouse anterior insula suppresses interest in social novelty by reducing spontaneous activity of excitatory neurons [38]. Likewise, depressive symptoms have been observed in rats after intracerebroventricular all-*trans* retinoic acid (ATRA) administration [39]. In addition, RARA is more abundant in CRH neurons of affective disorder hypothalami [40], where it both upregulates corticotropin-releasing hormone (*CRH*) expression and blocks glucocorticoid negative feedback on *CRH* [41], suggesting a link between retinoid TFs and elevated hypothalamic-pituitary-adrenal axis activity in MDD. Finally, given the substantial shared genetic risk across psychiatric disorders [42], it is notable that schizophrenia GWAS loci show enrichment for retinoid TR [43], and that circulating retinoids are dysregulated in schizophrenia patients [44]. Similarly, retinoid pathway genes, including *CYP26B1*, are dysregulated in postmortem brain from autism spectrum disorders, bipolar disorder, and schizophrenia patients [45]. Interestingly, retinoid deficiencies have been associated with these diseases, including recent observations of reduced serum levels of retinoic acid and its precursor, retinol, in schizophrenia [44]; similarly, reductions in serum retinol and expression of all three *RAR* genes were shown in autism spectrum disorders [46]. These findings led us to speculate that a component of MDD-associated genetic risk may likewise demonstrate an upstream convergence via recurrent retinoid-mediated TR disruptions across loci.

Massively parallel reporter assays (MPRAs) provide a solution to both experimentally identify functional variants and, consequently, their shared TR features. MPRAs assess thousands of DNA elements for transcriptional-regulatory functions and allelic differences simultaneously by pairing each short genomic sequence element of interest to several unique barcodes, with a constant promoter and reporter gene placed in between [47–50]. Delivery of a library of DNA elements to cells, followed by RNA collection and sequencing, enables quantitative estimation of the expression driven by each element as a ratio of expressed RNA barcode to delivered DNA barcode. These assays have recently been adapted to systematically identify SNPs with functional allelic TR differences from GWAS loci for several diseases [51–59]. Two key features make MPRAs advantageous for identifying both functional SNPs and their TR interactions. First, the assay is carried out via transfection and targeted RNA sequencing, meaning it can be executed in unmodified cell lines appropriate to the application. Second, MPRAs can be conducted to define TR effects of experimental manipulations in these systems, such as drug administration [60, 61].

Therefore, we sought to experimentally identify functional TR SNPs from 39 GWAS loci associated with MDD, neuroticism, and broader psychiatric disease risk, with the hypothesis that functional SNPs converge at the level of retinoid-mediated TR. From broad linkage regions, we selected over 1000 SNPs based on overlapping human brain and neural epigenomic signals suggestive of TR activity. Critically, selection of neither the loci nor the SNPs was predicated on retinoid involvement, allowing for unbiased functional screening of a cross-section of MDD GWAS loci. To ensure we could detect SNPs subject to retinoid-mediated TR, we used neuroblastoma (N2a) cells, as they are strongly and rapidly retinoid-responsive [62, 63]. Our initial assay identified over 75 functional SNPs from 29 GWAS regions, confirming that GWAS loci contain several functional SNPs. We then examined whether these functional SNPs possessed shared upstream TR features—namely, transcription factor (TF) binding motifs. Remarkably, there was indeed enrichment of retinoic acid binding TFs among the MPRA-functional vs. -non-functional SNPs, supporting our hypothesis. To further characterize retinoid effects on TR at MDD-associated SNPs, we performed a second assay using all-*trans* retinoic acid (ATRA), known to stall division of N2a and other neuroblastoma cells by inducing neuronal-like differentiation [62]. First, we found that functional SNPs containing retinoid receptor motifs had increased magnitudes of effect in the presence of ATRA, consistent with bonafide retinoid receptor TR activity. More broadly, ATRA led to striking rearrangements of the baseline regulatory landscape, including altered magnitude and reversed direction of allelic effects. In addition, it revealed new SNPs with allelic TR differences unmasked by ATRA treatment. Significant ATRA-allele interaction SNPs largely overlapped RXRA binding sites from chromatin immunoprecipitation (ChIP)-seq, as well as motifs of several known retinoid-induced TFs, indicating broad roles of both retinoid TFs and their downstream TR systems at functional MDD-associated SNPs.

Finally, we explored the cell type-specificity of TFs predicted to regulate our functionally identified SNPs. Strikingly, we found TFs highly specific to serotonin neurons were strongly enriched among those we predicted to be recurrently involved in retinoid-dependent SNP function. These findings suggest that the broad transcriptional-regulatory systems engaged by retinoids—and as we illustrate, the genetic component of MDD risk *they* engage—may converge on serotonergic neurons. In summary, we identify MDD-associated functional SNPs with both baseline and ATRA-mediated allelic differences in TR, and these disproportionately show upstream convergent regulation by retinoid receptors and TFs they induce. This highlights a striking potential point of convergence between genetic risk loci and an environmental risk factor for MDD.

## Methods

### Identifying candidate psychiatric GWAS regulatory variants

**Fig. 1:**
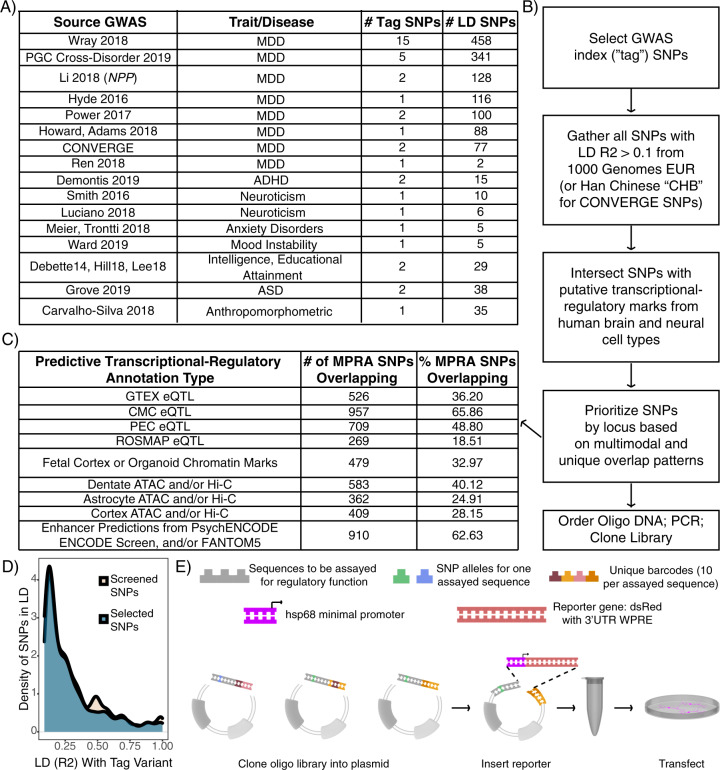
Design of an MPRA library to identify candidate functional SNPs in MDD loci. **A** Table of GWAS studies and number of loci covered in the MPRA library. **B** Flow chart of design and prioritization process. **C** Brain and neural transcriptional-regulatory predictive annotation overlap with SNPs included in MPRA library. Fraction and number of SNPs in designed MPRA library intersecting each transcriptional-regulatory predictive annotation type. **D** The manual prioritization process was not LD biased. The subset of prioritized SNPs are spread over LD space similarly to the full set of screened SNPs. **E** Schematic of library construction and delivery. Panel adapted from [50].

To prioritize putative regulatory variants from neuropsychiatric disease GWAS regions (predominantly MDD; Fig. 1A), SNPs in linkage disequilibrium (LD) with GWAS tag variants at *R*^2^ > 0.1 were collected and intersected with histone modification, eQTL, Hi-C, and enhancer segmentation datasets from human postmortem tissue and neural lineage cell lines (see Supplemental Methods, Fig. 1B). SNPs were manually selected based on diversity and density of annotation overlap within each locus (Supplemental Methods). As a negative control, we identified candidates from one additional locus associated with several anthropomorphic traits [64], in a trait-blinded manner. Altogether, 1453 SNPs were selected. Final LD of selected SNPs was distributed similarly to starting SNPs (Fig. 1D). To confirm that we could detect CNS-relevant regulatory SNPs, a positive control TR SNP functionally demonstrated in mouse retina and brain [57] was also included.

Human genomic sequence (hg19) tiles up to 126 bp were taken centered on the 1454 candidate enhancer SNPs, each paired to ten unique 10 bp barcode sequences per allele and ordered as an oligonucleotide (oligo) pool from Twist Bioscience (San Francisco, CA). Also in the pool were 110 “basal” barcodes (no human genomic sequence cloned upstream of the minimal promoter), such that the only variable sequence between reporter clones was the barcode itself. The oligos were PCR amplified, then cloned into plasmid (Fig. 1E); subsequently, a reporter cassette containing a minimal promoter (*hsp68*) driving the dsRed reporter gene [65] and the untranslated “woodchuck” element (for RNA stabilization, to improve signal) [66] was cloned in.

### Massively parallel reporter assays

N2A cells were grown in uncoated 6-well plates in medium consisting of 0.1 µM vacuum-filtered DMEM with 10% Fetal Bovine Serum (2% fetal bovine serum for the ATRA assay, based on media conditions from the literature [67, 68]). For transfection, cells were reverse transfected by plating in antibiotic-free medium onto pre-plated 400 µL mixtures of 2.5 µg plasmid with Lipofectamine 2000. In the first assay, *n* = 6 replicate wells were transfected and co-prepared for sequencing. A power analysis of these results using the 25th, 50th, and 75th percentile standard deviation of sequence expression measurements indicated we were ≥80% powered to detect Bonferroni-corrected p < 0.1 variant effects as low as abs(log2FC) 1.1 (*Supplemental Methods*). In the drug MPRA experiment, *n* = 12 wells were transfected, harvested, and prepared for sequencing together, with *n* = 6 ATRA-treated and *n* = 6 vehicle-treated.

After transfection, cells incubated for 7 h at 37 °C and 5% CO_2_. Medium was replaced with the respective medium containing antibiotics, and in the second assay, a final concentration of 20 µM ATRA dissolved in DMSO, or equivalent volume of vehicle (DMSO). Medium was not replaced before RNA collection in the first assay; in the second assay, it was refreshed every 24 h. Seventy-two hours after transfection, cells were collected and RNA extracted using the Zymo (Irvine, CA) Clean-and-Concentrator 5 kit per manufacturer instructions. Eluted RNA was treated with Turbo DNA-free kit to remove any residual plasmid to prevent contaminating DNA reads during sequencing, and cleaned a second time using the Zymo kit as above.

### Targeted sequencing of RNA and input plasmid

Briefly, equal amounts of RNA (1 µg) from each sample were prepared for sequencing by targeted cDNA synthesis using a primer against the distal 3′UTR of the reporter. These, along with input plasmid, were subjected to PCR, enzymatic digestion, ligation of Illumina sequencing adapters, and a final PCR to add sample indexes for sequencing. Enzymes, and size-selection cleanup steps used in this process are fully detailed in Supplemental Methods. No-reverse-transcriptase controls utilizing sample RNA were co-prepared for both experiments and did not generate detectable product, indicating sequencing amplicons generated from RNA samples were exclusively representative of RNA content. Samples were sequenced to an average depth of ~8 million reads (first assay) or ~20 million reads (second assay).

### Analysis

Allelic SNP effects on expression in the first assay and in single-condition analyses of the second assay were assessed by *t*-testing the element’s expression of each allele across replicates. In the first assay, over 90% of SNPs had normally distributed expression values (Shapiro–Wilk test, *p* > 0.05). Uncorrected *t*-test *p*-values and Mann–Whitney U test *p*-values were well-correlated for the 89 non-normally distributed SNPs (Pearson’s *r* = 0.825). Nonetheless, for *t*-test significant SNPs P_emp_ not passing the Shapiro–Wilk test, we verified the result by checking for a nominally significant Mann–Whitney U test at *p* < 0.05. No SNPs were excluded from analysis on this basis. dbSNP-assigned reference (“ref”) and alternative (“alt”) alleles for each SNP were used to define comparison direction (the difference of activity under the alt allele vs. the ref allele). For the first MPRA and single-condition analysis of vehicle samples from the second assay, empirical *p* values (Pemp) were calculated via simulated allelic comparisons between random subsets of “basal” barcodes (see Supplemental Methods) following an analogous procedure from a multiplex CRISPR study [69], with significance defined as Pemp < 0.05 unless specified otherwise. This ensures that a representative cross-section of expression variability driven by barcode sequences is accounted for when assessing TR differences. Single-condition analysis of ATRA samples utilized standard Benjamini–Hochberg FDR correction, as primary effects of interest in these samples were ascertained by linear modeling. For analysis of ATRA effects, we verified that variances were similar between the drug and vehicle conditions; indeed, the median barcode expression level standard deviation was 0.1216 in ATRA-treated and 0.1226 in vehicle-treated samples (with respective 25th and 75th percentile standard deviations also matched within 0.005 expression units). We calculated samplewise barcode-level expression values passing the “single-condition” filtering steps used for *t*-testing (Supplemental Methods) were fitted using a linear mixed model (LMM) requiring a minimum of 40% (96) of the 240 possible expression measurements per SNP. The LMM included a random term for replicate (to account for well-specific effects), expressed as: barcode expression ~ allele + drug + allele:drug+ (1|replicate). Empirical *p* value calculation from LMM *F* statistics was performed in an analogous manner to the prior experiment, generating a vector of F statistics for each coefficient from 20,000 randomized basal-only comparisons. All SNPs with an interaction Pemp < 0.05 also had a likelihood ratio test (LRT) *p* < 0.051 comparing a maximum-likelihood (ML) interaction model to an ML LMM with additive terms only, indicating that the interactive model was more predictive but not overfit compared to an additive model. For SNPs with significant allele and interaction coefficients, a meaningful allele main effect was considered present if the single-condition vehicle and ATRA analyses showed the same allelic direction of effect, with a vehicle Pemp < 0.1 and ATRA FDR < 0.1 (i.e., near-significant within each condition of *n* = 6, thus reasonably capable of achieving significance in the LMM analysis of the two conditions combined).

### MotifbreakR analysis and functional SNP enrichment for perturbed motifs

The motifbreakR [70] package was used to identify TF binding motifs significantly different between alleles of each SNP. Briefly, the number of MPRA-identified functional SNPs matching a given TF’s motif(s) for at least one allele was compared to the number of non-functional SNPs matching across 10,000 random draws of *n* (number of significant) SNPs. A second version of this analysis focused on the concordance rate—that is, whether the frequency of functional variants experiencing concurrent strengthening of motif and expression or vice versa—was significant compared to 10,000 draws of *n* random SNPs from the analyzed set. Analysis of the first assay’s SNPs defined functionality based on a P_emp_ threshold of 0.05. We performed two motif analyses of the second assay results, one comparing allele main effect SNPs (Pemp < 0.1) to those with Pemp > 0.1 for allele, drug, and interaction effects, representing the breadth of functional variant-susceptible *cis*-regulators. The second analysis compared interaction SNPs (Pemp < 0.05) to SNPs with an allele main effect (Pemp_allele _< 0.1) but no interaction (Pemp_interaction_ > 0.1).

### Analyses of functional-SNP enriched TF expression in human brain and chromatin immunoprecipitation (ChIP)-seq

We utilized outside ChIP-seq datasets to validate motif-based predictions of retinoid receptor binding and refine prediction of involved TFs. We intersected our functional SNPs to 25 tracks of ChIP-seq for retinoid receptors (19 human [71, 72], 6 mouse (3 ATRA-treated, 3 vehicle-treated) converted to hg19 coordinates using UCSC’s LiftOver [73]); 11 tracks of RXR-heterodimerization partners (10 human THRA/THRB [71, 72] and one aggregate analysis of human VDR [74]); and human genome-wide predictions of DR5 [75], a canonical RAR•RXR heterodimer binding sequence. For functional SNPs implicated at an RAR, RXR, VDR, or THRA/B site by either motifbreakR or ChIP, we identified potential target genes using chromatin-conformation [76] and eQTL [77–80] data. We performed broad-scope gene enrichment analysis of this gene set using Enrichr [81]. To examine shared biology of TFs implicated by motifbreakR enrichment at functional variants, we utilized PantherDb [82]. We finally examined TFs for enrichment among highly-expressed genes in adult and developing human brain using the ABAEnrichment package’s Wilcoxon approach [83], effectively weighting TFs by the number of functional SNPs implicated by motifbreakR (see Supplemental Methods).

## Results

### Many MDD loci contain more than one functional SNP

We identified >1000 SNPs from MDD-associated GWAS loci, prioritizing SNPs overlapping with epigenetic data from neural samples, and cloned them into an MPRA library (Fig. 1). We included one positive control SNP, shown to alter neural tissue gene expression, and one control locus near *CDKAL1* not a priori associated with psychiatric disease. To identify functional variants from these SNPs, the library was transfected into N2a cells (*n* = 6 replicates, Fig. 1E). Variant activity was assessed by RNA sequencing and barcode counts compared to input plasmid barcode counts. After filtering for read depth and barcode representation, 1013 SNPs spanning all 40 LD regions remained for analysis. Results were highly replicable across samples (Pearson *r* 0.63–0.85 for barcode expression; 0.90–0.96 for elements, Supplemental Figure S1). We use “element” to signify the set of barcodes corresponding to one unique sequence of interest (1 SNP = 2 elements).

**Fig. 2:**
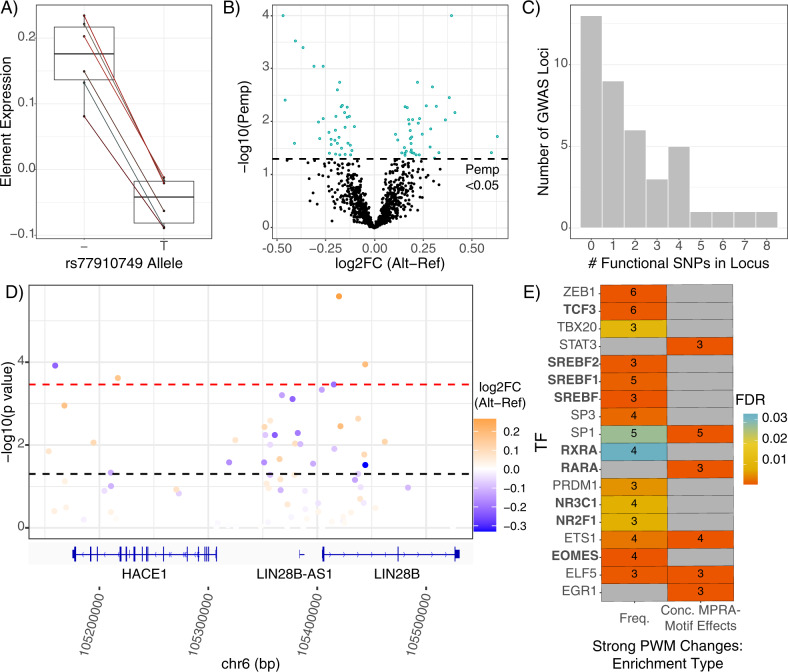
MPRA defines SNPs with a functional effect on gene expression. **A** MPRA results of positive control SNP. Shen et al. found that the T allele drove decreased expression relative to the deletion (“–”) allele, which was robustly reproduced in the present assay. **B** Volcano plot of allelic differences in reporter expression. Points represent one SNP’s composite log2 allelic fold change (alt vs. ref), determined as the mean of samplewise alternative allele barcode expression minus the matched mean of reference allele barcodes. The dotted line indicates the statistically corrected significance threshold. **C** Number of functional SNPs (MPRA-significant SNPs) per GWAS locus in the assay. Number of loci (*y*-axis) containing a given number of MPRA-significant (Pemp < 0.05) SNPs (*x*-axis). **D** The LIN28B locus harbors several functional SNPs. SNPs are plotted according to their chromosomal position (hg19) and colored based on their composite log2 allelic fold change. Refseq genes are visualized by the Integrative Genomics Viewer [116]. **E** TF binding motifs involved in retinoid signaling, steroid synthesis and response, and neural activity are enriched among functional SNPs. Boxes are colored by FDR-corrected significance of enrichment for motifbreakR-defined “strong” allelic perturbations to binding motifs among functional SNPs; the number of functional SNPs perturbing (left column) and/or with concordant motif and MPRA effects (right column) are shown. Concordant effects were defined by greater MPRA expression driven by the allele better-matched to the corresponding TF motif and vice versa—the expected behavior of strictly activating TFs.

Of 1013 SNPs analyzed, we identified significant allelic TR (Pemp < 0.05) at 76 SNPs (65 from MDD loci; 1 from the control *CDKAL1* locus) across 27 of the 40 analyzed GWAS regions, with effects ranging 0.1 to 0.63 (median 0.2) log2 fold-change (Fig. 2B). Interestingly, the functional variant from the control locus is suggestively associated (GWAS *p* < 5 × 10^−6^) with *“Poisoning by analgesics, antipyretics, and antirheumatics”* in UK Biobank [84]. As this likely includes attempted suicides, the SNP was retained for analyses. The positive control SNP, which we utilized to confirm our ability to detect small-effect sizes expected of regulatory SNPs, showed the expected lower expression of the T allele at a Pemp of <0.051 (Fig. 2A) [57].

While our assay was designed to broadly examine wide LD regions around GWAS index variants, we did identify one functional variant, rs11209952, in a fine-mapped credible set of variants for seeking general practitioner care for depression in UK Biobank [85]. Moreover, consistent with prior studies of “conditional” or “secondary” SNP associations—wherein additional LD SNPs have associations independent of their linked, larger-effect variant [20, 86, 87]—we identified several loci with multiple functional SNPs (Fig. 2C) (range 1–8, mean 2.8, median 2). Notably, we identified as functional rs1806153, a recently defined “conditional SNP” for MDD [20]. Our findings support models predicting multiple functional SNPs in GWAS loci, and directly validate one such finding from association analysis.

One notable TR SNP we identified, rs314267, comes from a “*LIN28B*” (nearest gene) GWAS locus repeatedly linked to MDD [9, 12] as well as cross-psychiatric disorder risk [42]. MPRA significance and effect size are illustrated for the region, showing that this locus contains several functional SNPs (Fig. 2D). All significant MPRA SNPs in the locus had effect directions consistent with brain eQTLs. rs314267 is the most significant *LIN28B* eQTL SNP (eSNP) in the region in PsychENCODE [78], and is a CommonMind Consortium (CMC) eSNP for both *LIN28B* and *HACE1* [77]. *HACE1* is also downregulated in postmortem MDD hippocampal CA1 [88]. Hi-C data from human neural cell cultures suggest rs314267 is within a neuron-specific *LIN28B* regulator, with promoter chromatin contacts found in dentate and cortical neurons, but not astrocytes [76]. *LIN28B* plays broad roles in neurodevelopment [89] and has potentially sex-differentiated functions [90–93]; considering sex differences in MDD prevalence and severity [94, 95], *LIN28B* constitutes an especially interesting gene target from this locus. Finally, we examined potential upstream TR mechanisms for SNP activity using VARAdb [96]. Query of rs314267 revealed a two order of magnitude allelic difference in the motif match *p*-value for *TCF4*—a gene itself implicated in cross-psychiatric-disorder risk [42, 97]. Overall, the identification of functional SNPs implicated in regulation of *HACE1* and *LIN28B* exemplifies the ability of MPRAs to identify functional variants involving sensible TR mechanisms and target genes.

### Shared regulatory architecture across distinct loci

We next sought to test our hypothesis that functional MDD risk variants shared retinoic acid-related TR architecture. If so, functional SNPs should disproportionately disrupt binding sites of retinoid-binding TFs compared to SNPs without an allelic effect on TR. Such data would indicate that MDD risk is mediated in part through perturbations of specific upstream transcriptional circuits and may highlight how risk conferred through retinoids converges with risk conferred through genetics to perturb downstream gene expression.

To take an unbiased approach to our retinoid hypothesis, we broadly analyzed all motifs showing enrichment at TR SNPs. Motifs for several dozen TFs were perturbed by the functional SNPs more frequently than expected, often with ‘strong’ perturbations to motifs and/or overrepresentation of concordant expression effects (Fig. 2E, Supplemental Figure S2). This included several TFs aligned with biological processes relevant to psychiatric disease. For example, several TFs are involved in steroid pathways, from regulating biogenesis (*SREBF* family, 6 SNPs) to conveying downstream TR effects—most notably, via glucocorticoid receptor (*NR3C1*, 5 SNPs; Supplemental Figure S3), a central component of the stress response. Functional SNP overrepresentation of SREBF motifs is consistent with high expression of these TFs in N2as and related neuroblastomas [98, 99]. A second group of transcription factors included three TFs involved in neural lineage commitment/development: TCF3 [100, 101], EOMES, and NR2F1 [102] (6, 4, and 3 SNPs, respectively). Altogether, functional SNP enrichment for these TFs’ motifs bolster our confidence in this approach, as (a) detected variation involves TFs known to be expressed in N2As (*SREBF*); (b) functional variation involves TFs with roles in developing CNS, where disease variants likely act; and (c) that the single-best characterized trigger of MDD (stress) is reflected in enrichment of alterations to NR3C1 motifs.

**Fig. 3:**
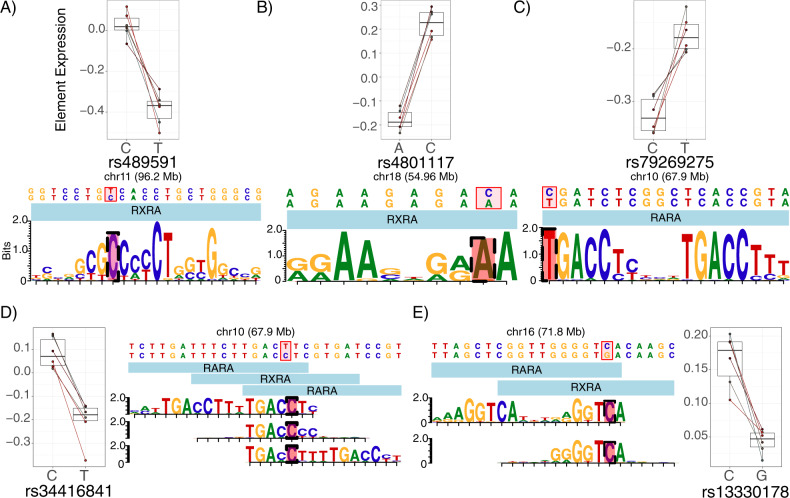
MPRA signal at SNPs disrupting putative retinoid TF motifs. The five functional SNPs driving enrichment signals for retinoid TF motif perturbations are shown, along with the MPRA results for each variant. Each motif diagram shows only distinct position-weight matrices (PWMs) (motifbreakR uses a large meta-collection of motifs, which were often identical or nearly identical across retinoid TFs; such redundant motifs are not shown). Among functional-SNP enriched retinoid TFs, **A** rs489591 and **B** rs4801117 exclusively perturb RXRA motifs. **C** rs79269275 perturbs an RARA (or RARB, identical but not shown) motif. **D** rs34416841 alters several similar retinoid motifs across multiple positions and TFs. Not shown: near-identical motifs for RARB along the same sequence as the 5′ RARA motif; near-identical RXRB and RARB motifs along the same sequence as the center RXRA motif; and a near-identical RARB motif along the same sequence as the 3′ RARA motif. **E** rs13330178 disrupts an RARA or RXRA binding site. Given that RXRA and RARs are known to heterodimerize, it is possible that this SNP disrupts the RXRA component of such a heteromer’s binding sequence.

Finally, consistent with our hypothesis of convergence on retinoid-mediated TR, functional variants were enriched for “strong” perturbations of retinoid receptor TF motifs (Fig. 3), including RARA, RARB, and RXRA (5 SNPs from 4 MDD loci, Fig. 3). Especially notable is the motif configuration at SNP rs34416841, which falls within three partially overlapping motifs for retinoid TFs. In addition, the elements overlapping rs489591 and rs13330178 appear to be functional human retinoid TF binding sites in vivo based on DNAse hypersensitivity footprinting [103].

### Retinoids unmask additional functional SNPs in MDD loci

Our findings supported the hypothesis that MDD-associated variants across multiple loci converge on TR, including that modulated by retinoids. We thus designed a pharmacological follow-up with two goals in mind. The first goal was to functionally verify that retinoids were involved in TR at SNPs where their motifs were found (in *cis*), and potentially unmask additional retinoid-targeted alleles. Our second goal was to further assess retinoid signaling *trans* (i.e., indirect) effects on variants from these same GWAS regions, e.g., via non-retinoid TF induction, co-regulation, or repression [33]. Therefore, we performed a second MPRA with an all-*trans* retinoic acid (ATRA) condition.

**Fig. 4:**
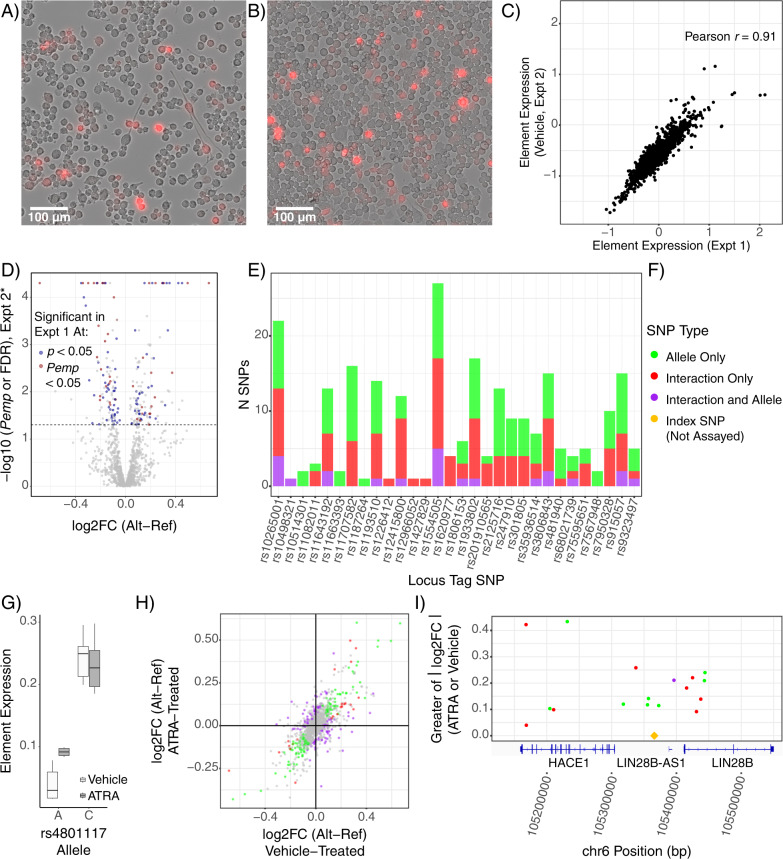
Retinoid treatment alters transcriptional regulation and unmasks additional functional variants. **A** ATRA-treated cells show growth arrest and neurite growth, demonstrating effective ATRA treatment, while **B** vehicle-treated cells continued to proliferate in a de-differentiated state. **C** Results of the vehicle treatment replicate the initial MPRA findings. Element (single-allele) expression values for each sequence assessed in both assays is plotted. **D** Significant and marginally significant functional SNPs from the first assay showed effects in the second assay. The larger allelic difference value from the ATRA and vehicle single-condition analyses is plotted for each SNP on the *x*-axis; the *y*-axis value is the corresponding corrected *p*-value (FDR correction for the ATRA-only analysis or P_emp_ for vehicle-only analysis). **E** Retinoids unmask functional SNPs with additional or exclusive retinoid-mediated effects. **F** SNP effect(s) color key for panels (**E**), (**H**), and (**I**). SNPs with both effects were those with LMM interaction Pemp < 0.05, LMM allele Pemp < 0.05, and both single-condition analyses showing the same allelic effect directionality at ATRA FDR < 0.1 *AND* vehicle P_emp_. **G** rs4801117-A shows greater activity with ATRA treatment while the C allele is unaffected. The ATRA having an expression effect only on the A allele is consistent with the A allele matching the RXRA motif as shown in Fig. 3B. **H** Transcriptional-regulatory SNPs show a wide range of altered and unaltered effects with ATRA treatment. Single-condition log2FC values are shown. **I** Several additional SNPs with retinoid-dependent function (i.e., allele-ATRA interaction) in the *LIN28B* locus. Only significant SNPs are illustrated. Notably, there are several functional SNPs clustered around the GWAS index SNP, suggesting association signal at this locus may be driven by multiple functional/conditionally functional variants.

After 48 h, cultures were imaged to verify drug activity (as ATRA is light-sensitive) based on known morphologic responses of N2as to ATRA, which include neurite outgrowth and mitotic arrest [62, 104, 105]. Indeed, drug-treated cells had a qualitatively lower cell density and produced neurite-like processes (Fig. 4A) in comparison to vehicle-treated cells (Fig. 4B). After RNA sequencing, we first analyzed vehicle-treated replicates alone to ensure replicability of the assay. Element expression levels in the vehicle condition strongly correlated to the first experiment (Pearson *r* = 0.91; Fig. 4C), and replicated the functional variants (Fig. 4D); all 31 shared significant SNPs showed consistent directions of effect.

We next applied a linear mixed model (LMM) to identify SNPs responding to ATRA (that is, allele-drug interactions). A total of 1079 SNPs were analyzed after filtering for read and barcode depth. In part due to the effective doubling in power to detect allelic effects with 12 replicates and the LMM approach, we now identified 137 variants with a main effect of allele (129 from MDD loci). Four of the five retinoid receptor motif-perturbing variants from the first assay passed filtering; all four of these variants again showed allelic main effects (all Pemp < 0.01), as did many other functional variants identified in the previous experiment (Fig. 4D). To our surprise, more variants showed a significant drug-allele interaction effect: a total of 128 SNPs (122 from MDD loci) (Fig. 4E, F). Among the drug-allele interaction SNPs were one of the four retinoid-related SNPs identified from the first assay (rs4801117; Pemp_interaction_ < 0.025, Fig. 4G), while another trended toward interaction (rs489591; Pemp_interaction_ = 0.117). This strongly supports a role of retinoid TF activity at rs4801117 as predicted by the motif analysis. More broadly, comparison of changes between the two conditions reveals the striking extent to which the regulatory landscape of the N2As was altered by ATRA (Fig. 4F and H). Notably, several additional functional variants were identified in the previously highlighted *LIN28B* locus, further illustrating multi-variant and context-dependent aspects of GWAS loci (Fig. 4E and I). In all, this experiment highlights the ability of MPRAs to detect contextual influences such as cell states and signaling on functional noncoding variation, and to unmask distinct, context-dependent functional SNPs.

### Retinoids reveal additional axes of convergent regulation at functional MDD-associated SNPs at levels of TF and cell type

**Fig. 5:**
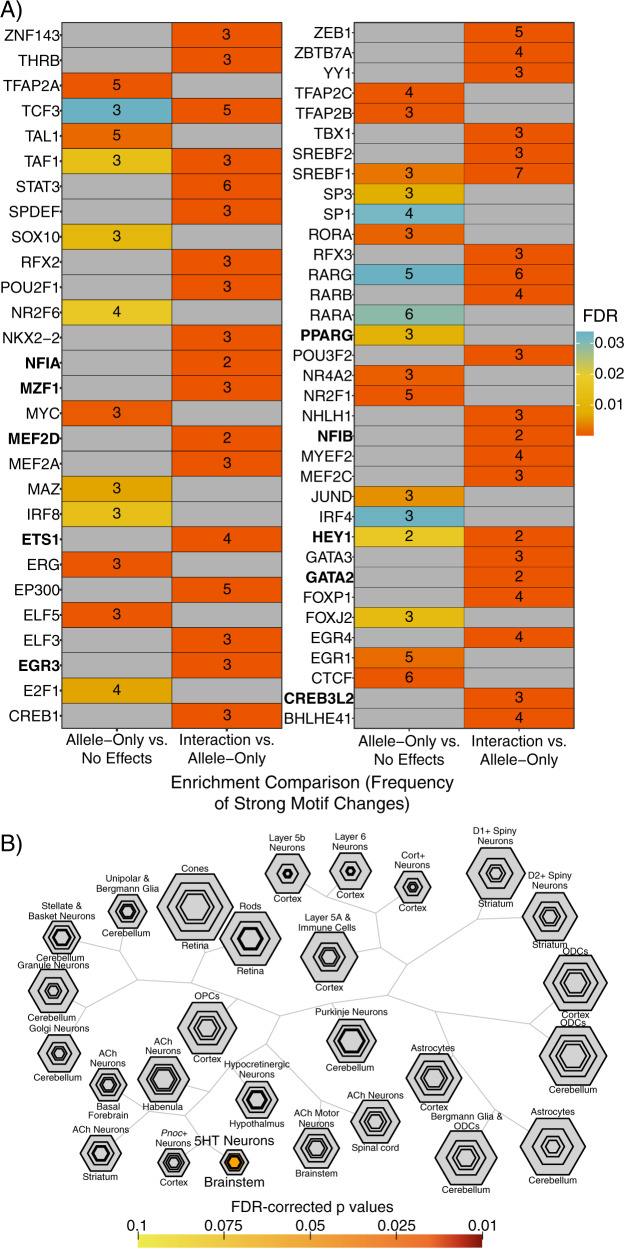
Distinct TFs underlying retinoid-dependent functional SNPs and implication of serotonergic neurons. **A** Motifs overrepresented among ATRA-independent (allele effect without interaction) SNPs (left columns) or among ATRA-dependent (interaction) SNPs (right column). The heatmap is shown in halves for visibility. TFs identified as ATRA-upregulated in human neuroblastoma lines [104] are in bold font. **B** TFs implicated by ATRA-interacting SNPs significantly overlap TFs enriched in serotonergic neurons. Plot generated using the cell-specific expression analysis (CSEA) tool (http://genetics.wustl.edu/jdlab/csea-tool-2/) [32]. 5HT serotonin, ACh acetylcholine, ODC oligodendrocyte, OPC oligodendrocyte progenitor.

As the ATRA-based assay provided improved power to identify allelic variant effects on expression, we again employed our motifbreakR-based analyses to assess convergent transcriptional mechanisms underlying identified regulatory variants. When examining SNPs with allelic effects in comparison to SNPs with no allelic, drug, or interaction effects, several retinoid receptor motifs were again overrepresented, including those of RXRA, RXRB, RARA, and RARG (Fig. 5A, Supplemental Figure S4), totaling 11 of the 92 allele main effect SNPs analyzed, spanning 10 MDD GWAS loci. These findings further support retinoid receptor binding sites as an upstream regulatory system recurrently involved in MDD risk genetics.

As retinoids resulted in stark changes across the transcriptional-regulatory landscape, we further sought to predict TFs potentially underlying allelic effects following retinoid exposure. Therefore, we also analyzed the interaction SNPs in comparison to allelic SNPs that were *not* subject to interactions. This revealed a novel set of TFs not observed in the preceding analyses, including TFs with roles in neural differentiation and maturation (Fig. 5A, Supplemental Figure S4), as well thyroid hormone receptor THRB, an RXR binding partner. We compared the overrepresented motifs to TFs recently demonstrated to be upregulated in human neuroblastoma lines (KCNR, LAN5) by ATRA. Of the 26 TFs identified as ATRA-induced in these two lines, motifs were available for 18 in our analysis. Of these, 6 of the TFs were enriched among allele main-effect-only SNPs, while 12 of these TFs were enriched among the retinoid-allele interaction variants [104] (Fig. 5A, Supplemental Figure S4), supporting our predictions of TFs playing ATRA-dependent roles at functional SNPs.

### Integrative analysis of TF sets at functional variants: TF binding, spatiotemporal brain enrichment, and putative target genes

As retinoid receptors have highly redundant binding motifs, we sought to both validate motif-based implication of retinoid receptors and more finely identify the particular TFs binding at functional SNPs. We aggregated ChIP-seq data for RAR, RXR, and RXR-heterodimerization partners (VDR, THRA, THRB) and identified functional SNPs overlapping peaks for each TF. Altogether, 35 of our 277 functional SNPs from across the two assays were in at least one such binding site (Supplemental Table 1). 15/17 of the allele-ATRA interaction SNPs overlapped a ChIP peak for RXRA, suggesting RXRA may be the common mediator of the observed retinoid-dependent SNP effects.

We also performed Gene Ontology analysis of functional variant enriched TFs against a background of all TFs in the motifbreakR tool using PANTHERdb but found no detailed Biological Processes of note (Supplemental Table 2). We next sought to examine whether TFs enriched at functional variants in our motif analyses corresponded to particular spatiotemporal expression patterns in the brain. To favor the most broadly-implicated TFs, we utilized the ABAEnrichment package’s Wilcoxon analysis approach on the TF sets from the ATRA experiment using the number of motifbreakR SNPs as the TF gene “scores”. In this analysis, several brain regions across developmental stages were nominally enriched (family-wide error rates <0.05) in ATRA-dependent and -independent TF expression, with especially broad enrichment at high expression thresholds (≥90th percentile) in adolescent brain (Supplemental Table 3). This does not appear to be an artifact of the cell model, considering that neuroblastomas are arrested in a neural crest progenitor (i.e., pre-/peri-natal cell type) stage. If replicated in future studies with larger adolescent sample numbers, this may suggest that retinoid-mediated aspects of MDD genetic risk are especially active in the adolescent brain, perhaps contributing to frequent emergence of the disorder around this time.

We additionally utilized these sets of TFs as gene sets to investigate whether retinoid-dependent or -independent regulatory variants might be particularly active in certain cell types of the brain. We screened for enrichment of these TFs among genes with strong cell type-specific expression in brain as previously defined for over 20 cell type translatomes [32]. Three TFs (spanning 8 ATRA-interacting SNPs) were discovered to be highly specific to serotonin neurons (Fig. 5B): *GATA2, GATA3*, and *FEV*, while no cell type enrichments under FDR < 0.1 were noted for TFs linked to ATRA-independent variants. Supporting these findings, an enrichment analysis of putative target genes (implicated by brain eQTL or neural Hi-C) of SNPs in retinoid TF motifs or ChIP peaks (Supplemental Table 4) revealed 5 genes nominally enriched for high regional expression in rhombomere 9, which gives rise to medullary populations of serotonin neurons [106] (The full results can be explored at https://maayanlab.cloud/Enrichr/enrich?dataset=27d6db2a8510a90ed0d78e6b60c59287).

Using the R2 database (http://r2.amc.nl), we examined expression of *FEV*, *GATA2*, and *GATA3* TFs in 24 human neuroblastoma lines (GEO accession GSE28019), retinoic acid-treated human SH-SY5Y neuroblastoma cells [107], alongside human neural progenitors [108] and melanoma lines as comparators [109], confirming neuroblastomas strongly express all three of these TFs (Supplemental Figure S5). Single-cell RNA sequencing data from mouse brain confirms the specificity of these TFs, revealing that these TFs are only expressed in serotonergic, noradrenergic, peripheral autonomic, and midbrain inhibitory neurons—with all three expressed in serotonin neurons [110]. Furthermore, exogenous retinoids have been shown to lower circulating serotonin in humans [46] and to alter morphology of rat raphe neurons in slice culture [111], suggesting these neurons are retinoid responsive. We do not believe our finding is an artifact of the N2a system, as we could identify no evidence in the literature suggesting a serotonin-like identity of N2a cells with or without ATRA treatment. Altogether, these findings suggest serotonin neurons and closely related cell types [93] may be cellular points of convergence for several retinoid-mediated functional SNP effects on MDD risk.

## Discussion

To date, most functional investigations of SNPs in the context of psychiatric disorders have taken place in a low-throughput manner, such as single-variant classical reporter assays [21] or using CRISPR-Cas9 technology to edit limited positions for deep phenotyping [112]. Here, we leveraged MPRA to screen over 1000 SNPs from loci associated with MDD, related phenotypes, and broader psychiatric disease, demonstrating the utility of this technique for dissecting the functional regulatory architecture of psychiatric GWAS loci, and defining shared upstream regulatory features across loci.

In doing so, we identify over 100 SNPs with allelic effects on expression, with most coming from loci containing ≥2 functional SNPs. These data provide experimental support for the prediction that multiple SNPs with allelic effects exist within GWAS loci as put forth in polygenic/omnigenic theory literature. We further examined the omnigenic hypothesis’ more central prediction of regulatory convergence across loci. By examining the shared regulatory features (TF binding motifs) based on enrichment at functional SNPs, we were able to predict several TFs with TR activity recurrently altered across MDD-associated SNPs, highlighting retinoid receptors in particular.

Retinoids are encountered both exogenously (e.g., as ATRA in oncology, and as isotretinoin, carrying a black-box warning for suicidality) and endogenously, including during brain development. To investigate how SNP functions may be altered by retinoids, we repeated the assay with an ATRA condition. ATRA drastically rearranged the TR landscape of N2a cells, resulting in altered and novel allelic effects at over 100 SNPs and revealing ATRA-dependent mechanisms of function across 122 SNPs from 22 of 26 MDD GWAS loci assessed. Of 17 ATRA-interacting functional SNPs overlapping ChIP peaks for retinoid receptors, 15 overlapped ChIP sites of RXRA, (Supplemental Table 1b) suggesting it may be central in functional SNP activity at retinoid receptor binding sites in this system. Interestingly, single-cell epigenomics of human cortical cell types recently found RXRA motifs to be uniquely enriched in open chromatin of SST interneurons [113], a strong candidate cell type for MDD [114]. These findings suggest that retinoid receptors—RXRA in particular—merit mechanistic follow-up regarding TR differences at MDD-associated SNPs. Future work may be able to leverage biobank-level datasets to ascertain whether retinoid-interacting SNPs are overrepresented in retinoid-treated patients experiencing adverse psychiatric side effects. While data on endogenous retinoids, e.g., plasma values, are not currently available in large genotype-phenotype-health record cohorts like UK Biobank, future datasets may enable investigation of circulating retinoids and their interaction with genotype in cognitive and psychiatric phenotypes.

The methodologic requirements of high-throughput assays such as MPRAs bring inherent limitations to their results. The primary precaution in interpreting these results concerns cell type relevance. MPRAs are subject to the TR landscape of the cell type used. Neuroblastoma cells, including N2As, are derived from peripheral neural crest progenitors—though they can be differentiated into dopaminergic neurons [105] and commit to neuronal differentiation with ATRA [62, 68]—and were selected for these assays based on intact retinoid signaling rather than representing a disease cell type per se. On the other hand, the neural crest-derived autonomic nervous system has received little consideration (relative to brain) in psychiatric genetics of MDD despite the well-appreciated role of stress in depression. These data may form an interesting foundation for future study of autonomic effects of MDD genetic risk.

Still, we can broadly speculate on brain cell types implicated by our findings. A notable prior pharmacology MPRA cleverly tested gDNA fragments for regulatory activity over a time course of dexamethasone treatment, while collecting epigenomic data in the same cell type over the same time course to compare MPRA signal and endogenous genomic marks. They found that endogenous genomic regulatory elements with repressive marks or depleted of glucocorticoid receptor binding were oftentimes active and/or dexamethasone-differentially active when assayed on the MPRA plasmids. This suggests that the transcriptional-regulatory capacity of an MPRA is not constrained by the epigenome of the model cell, but rather by its expressed TFs [61]. As such, retinoid receptor-mediated SNP functions observed are not limited to sequences that would be active in the N2a genome; as such, it is entirely plausible that the observed effects also occur in retinoid-receptor expressing brain populations. Mouse nervous system single-cell RNA-seq suggests retinoid receptor expression is absent in brain glia, but robust in many neuron types [110]. Thus, we suspect the directly-mediated retinoid receptor SNP effects we observe may be neuron-specific. Future studies may be able to address the interesting question of differences in neuronal subtypes exhibiting functional SNP effects.

We find that principles of the omnigenic model appear to hold true for MDD risk genetics, including the presence of far more functional variants (a total of 277 SNPs with allelic and/or interaction effects of 1178 assessed across the two assays; Supplemental Table 1) than there were GWAS loci (i.e., tag SNPs). We find, interestingly, that functional SNPs form convergent subsets of upstream (transcription-regulatory) sequences and systems, which in turn have shared retinoid dependence and are collectively enriched in serotonin neurons via 8 ATRA-interacting functional SNPs in binding motifs of GATA2, GATA3, and FEV. It has previously been demonstrated that systemic administration of ATRA depletes serotonin by over 40% in the rat brain [115], supporting the serotonin system as a convergent target of retinoid-regulated pathways. As GWAS of MDD begins to explore severe, treatment-refractory cases [7], it will be interesting to see whether associated variation still shows such convergence, as treatment-resistant depression (generally, non-response to two or more classes of antidepressant) effectively signifies non-response to multiple serotonergic agents.

In all, we assessed the architecture of *cis-*regulatory variation in psychiatric disease risk loci, identifying at least one functional SNP in the majority of the 40 GWAS loci examined, largely corresponding to MDD-associated SNPs. Strikingly, retinoid receptor binding sites and TR systems subject to regulation by ATRA have a substantial impact on whether and how MDD-associated SNPs are functional. These findings constitute a robust experimental demonstration of the influence of physiological and environmental states on the molecular activities of disease-associated SNPs, and constitute a high-confidence set of MDD SNPs meriting deeper functional characterization of both their TR mechanisms and their environmental interactions.

## Supplementary information

Supplemental Methods and Legends

Supplemental Figure S1

Supplemental Figure S2

Supplemental Figure S3

Supplemental Figure S4

Supplemental Figure S5

Supplemental Table 1

Supplemental Table 2

Supplemental Table 3

Supplemental Table 4

## Data Availability

A summary spreadsheet of all significant SNPs identified in one or both assays, along with full analysis results, including barcode-wise expression in each sample, single-condition allelic effect tests, linear modeling results, and significantly enriched TFs in each of the comparisons executed, along with the code utilized to execute these analyses, is available at https://bitbucket.org/jdlabteam/n2a_atra_mdd_mpra_paper/src.

## Data availability

A summary spreadsheet of all significant SNPs identified in one or both assays, along with full analysis results, including barcode-wise expression in each sample, single-condition allelic effect tests, linear modeling results, and significantly enriched TFs in each of the comparisons executed is available at https://bitbucket.org/jdlabteam/n2a_atra_mdd_mpra_paper/src. Raw sequencing files are deposited in GEO under accession GSE167519. Supplemental information is available at Translational Psychiatry’s website.
