# Supplementary figures and images for: Transcriptional-regulatory convergence across functional MDD risk variants identified by massively parallel reporter assays
Source: Transl Psychiatry. 2021 Jul 22;11:403. doi: 10.1038/s41398-021-01493-6 (PMC8298436; doi:10.1038/s41398-021-01493-6)

A)

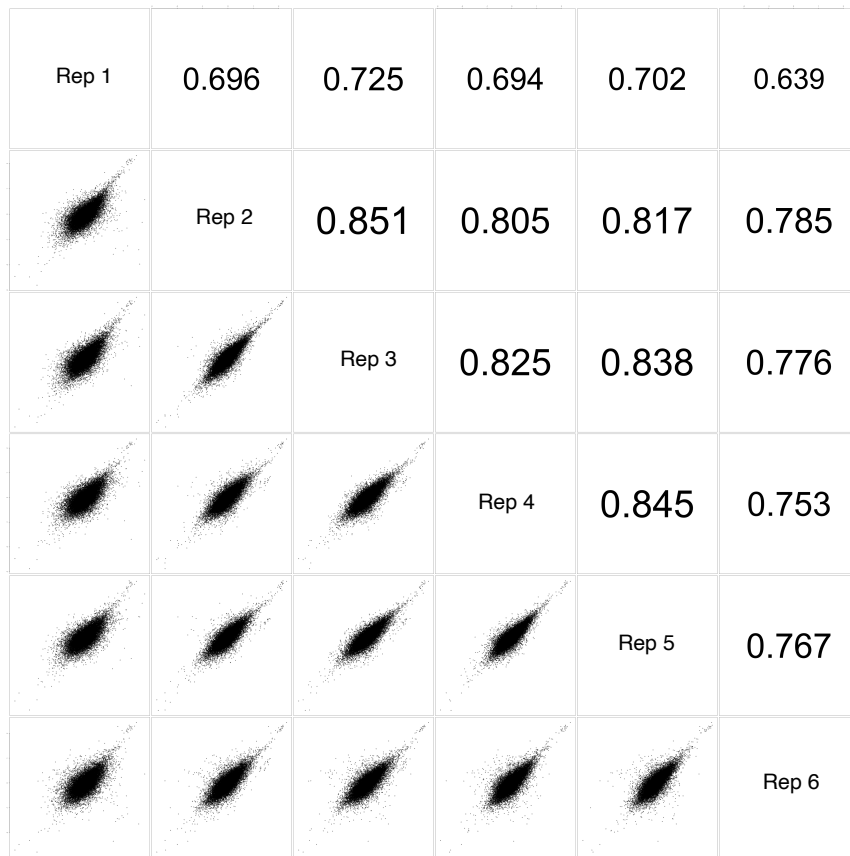

B)

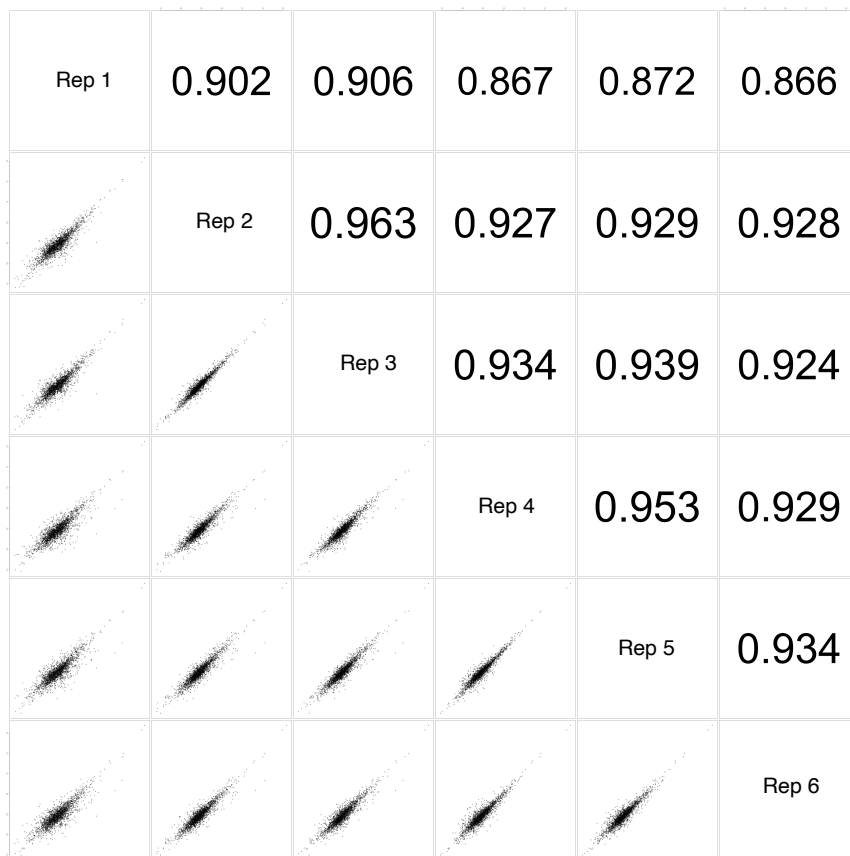

Supplement: Supplementary file 2 — Supplemental Figure S1 [file 41398_2021_1493_MOESM2_ESM.pdf]

A)

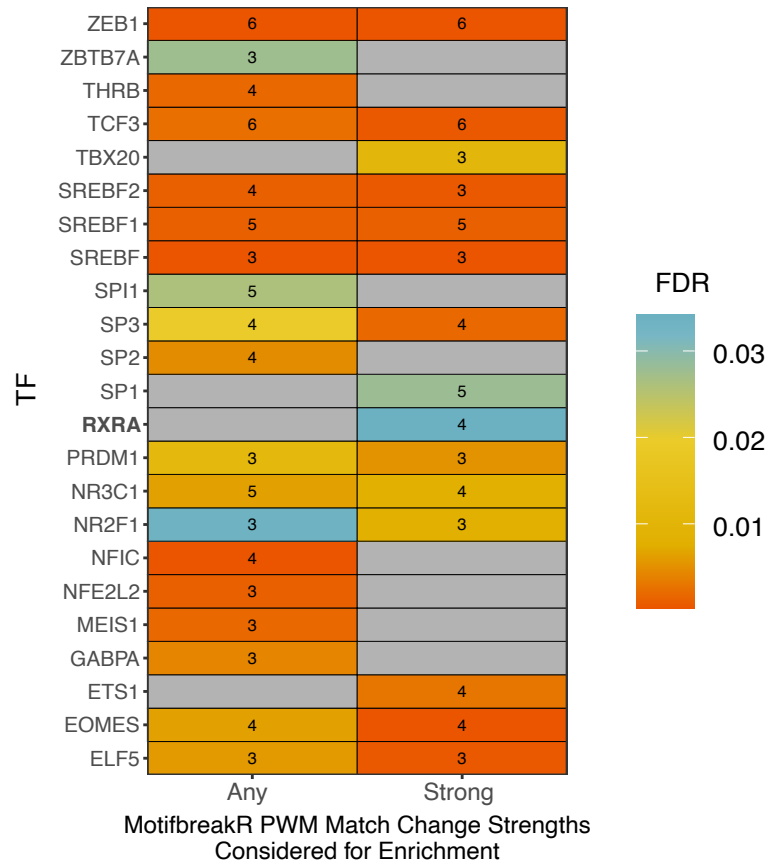

B)

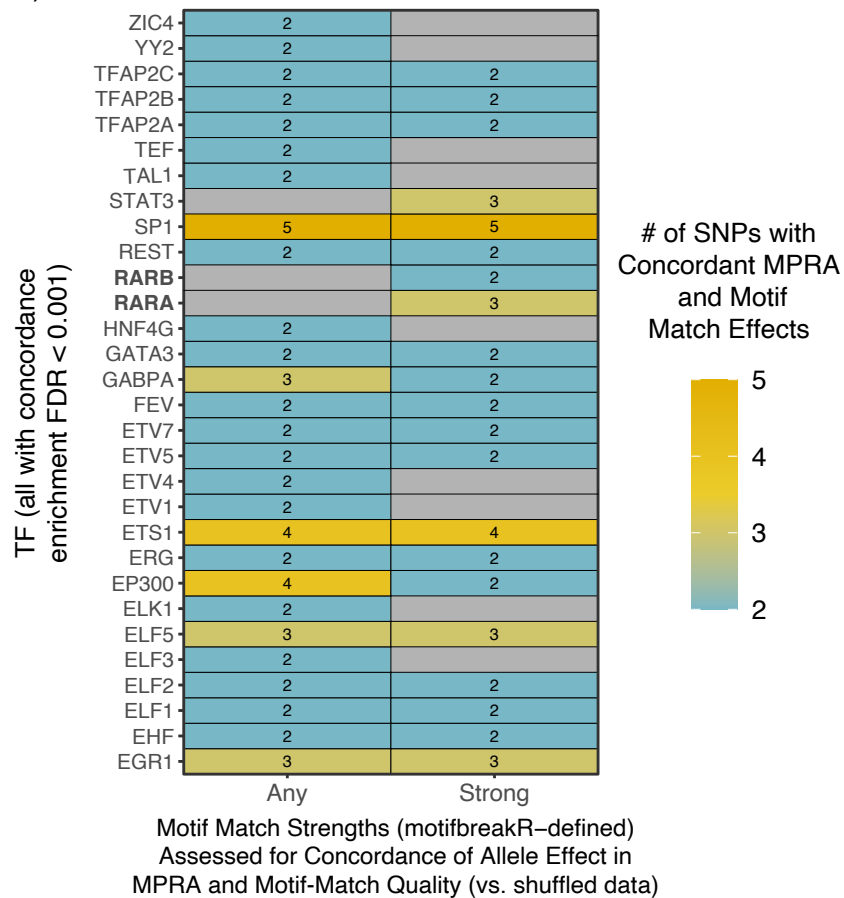

Supplement: Supplementary file 3 — Supplemental Figure S2 [file 41398_2021_1493_MOESM3_ESM.pdf]

A)

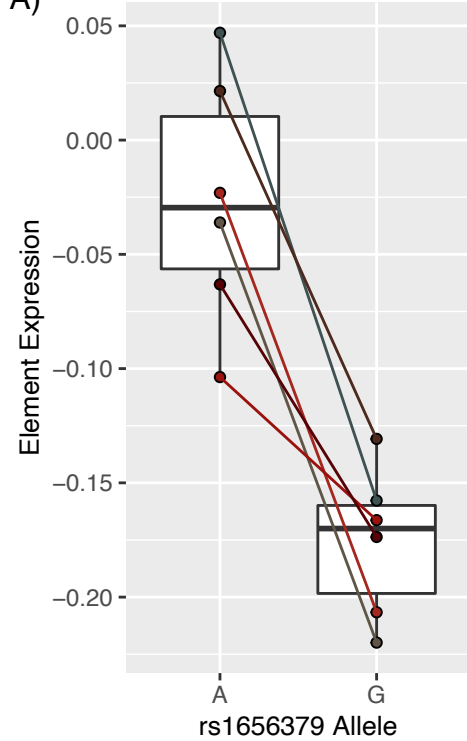

B)

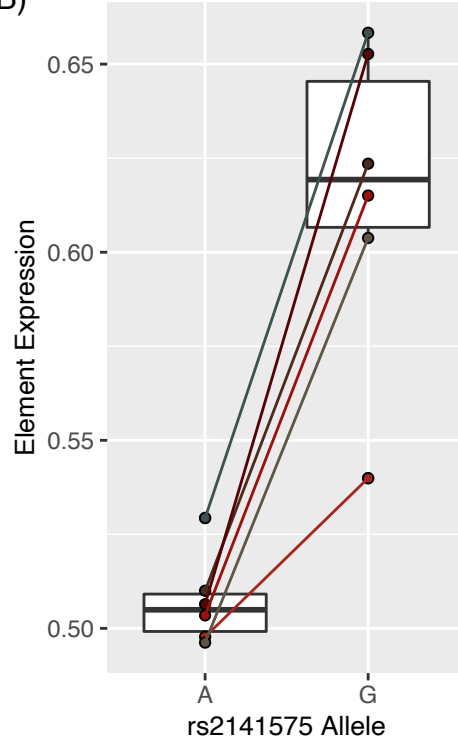

C)

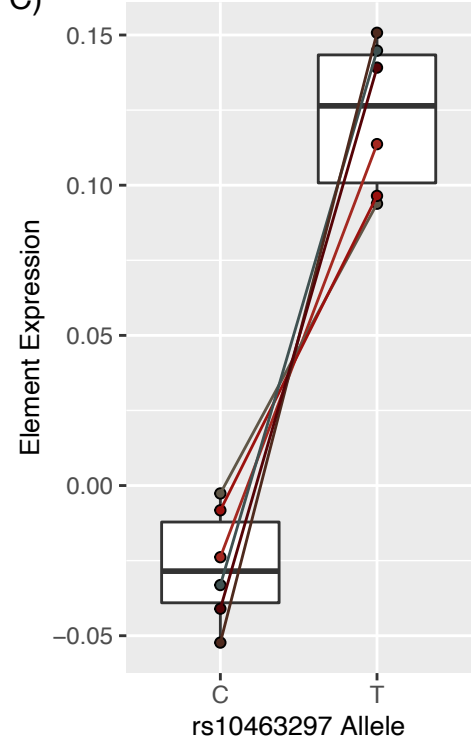

D)

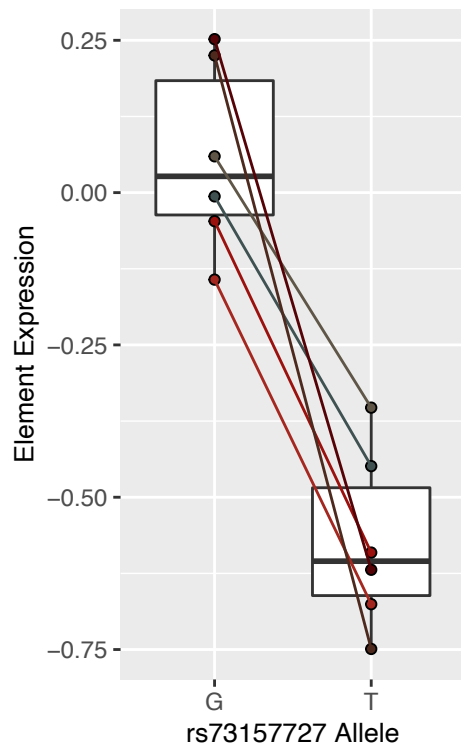

E)

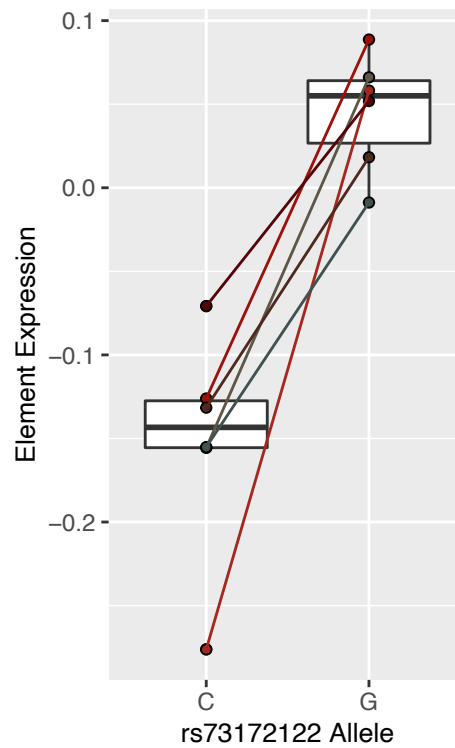

Supplement: Supplementary file 4 — Supplemental Figure S3 [file 41398_2021_1493_MOESM4_ESM.pdf]
