# Supplementary material for: Transcriptional-regulatory convergence across functional MDD risk variants identified by massively parallel reporter assays
Source: Transl Psychiatry. 2021 Jul 22;11:403. doi: 10.1038/s41398-021-01493-6 (PMC8298436; doi:10.1038/s41398-021-01493-6)
Supplement: Supplementary file 5 — Supplemental Figure S4 [file 41398_2021_1493_MOESM5_ESM.pdf]

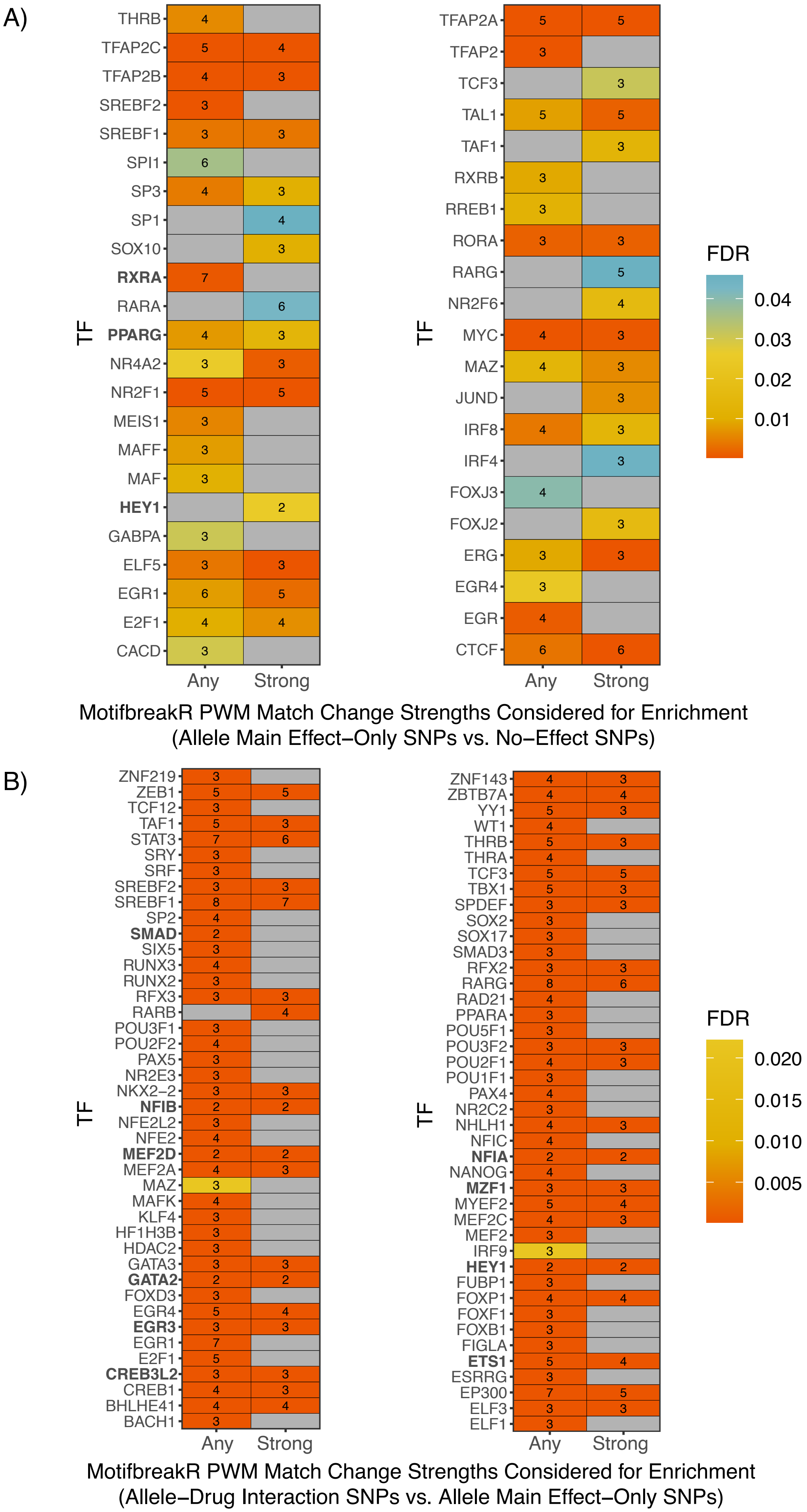
A)
THRB
TFAP2C
TFAP2B
SREBF2
SREBF1
SPI1
SP3
SP1
SOX10
RXRA
RARA
PPARG
NR4A2
NR2F1
MEIS1
MAFF
MAF
HEY1
GABPA
ELF5
EGR1
E2F1
CACD
TF
Any
Strong
TFAP2A
TFAP2
TCF3
TAL1
TAF1
RXRB
RREB1
RORA
RARG
NR2F6
MYC
MAZ
JUND
IRF8
IRF4
FOXJ3
FOXJ2
ERG
EGR4
EGR
CTCF
TF
Any
Strong
FDR
0.04
0.03
0.02
0.01
MotifbreakR PWM Match Change Strengths Considered for Enrichment
(Allele Main Effect−Only SNPs vs. No−Effect SNPs)
B)
ZNF219
ZEB1
TCF12
TAF1
STAT3
SRY
SRF
SREBF2
SREBF1
SP2
SMAD
SIX5
RUNX3
RUNX2
RFX3
RARB
POU3F1
POU2F2
PAX5
NR2E3
NKX2−2
NFIB
NFE2L2
NFE2
MEF2D
MEF2A
MAZ
MAFK
KLF4
HF1H3B
HDAC2
GATA3
GATA2
FOXD3
EGR4
EGR3
EGR1
E2F1
CREB3L2
CREB1
BHLHE41
BACH1
TF
Any
Strong
ZNF143
ZBTB7A
YY1
WT1
THRB
THRA
TCF3
TBX1
SPDEF
SOX2
SOX17
SMAD3
RFX2
RARG
RAD21
PPARA
POU5F1
POU3F2
POU2F1
POU1F1
PAX4
NR2C2
NHLH1
NFIC
NFIA
NANOG
MZF1
MYEF2
MEF2C
MEF2
IRF9
HEY1
FUBP1
FOXP1
FOXF1
FOXB1
FIGLA
ETS1
ESRRG
EP300
ELF3
ELF1
TF
Any
Strong
FDR
0.020
0.015
0.010
0.005
MotifbreakR PWM Match Change Strengths Considered for Enrichment
(Allele−Drug Interaction SNPs vs. Allele Main Effect−Only SNPs)
