# Supplementary material for: Transcriptional-regulatory convergence across functional MDD risk variants identified by massively parallel reporter assays
Source: Transl Psychiatry. 2021 Jul 22;11:403. doi: 10.1038/s41398-021-01493-6 (PMC8298436; doi:10.1038/s41398-021-01493-6)
Supplement: Supplementary file 6 — Supplemental Figure S5 [file 41398_2021_1493_MOESM6_ESM.pdf]

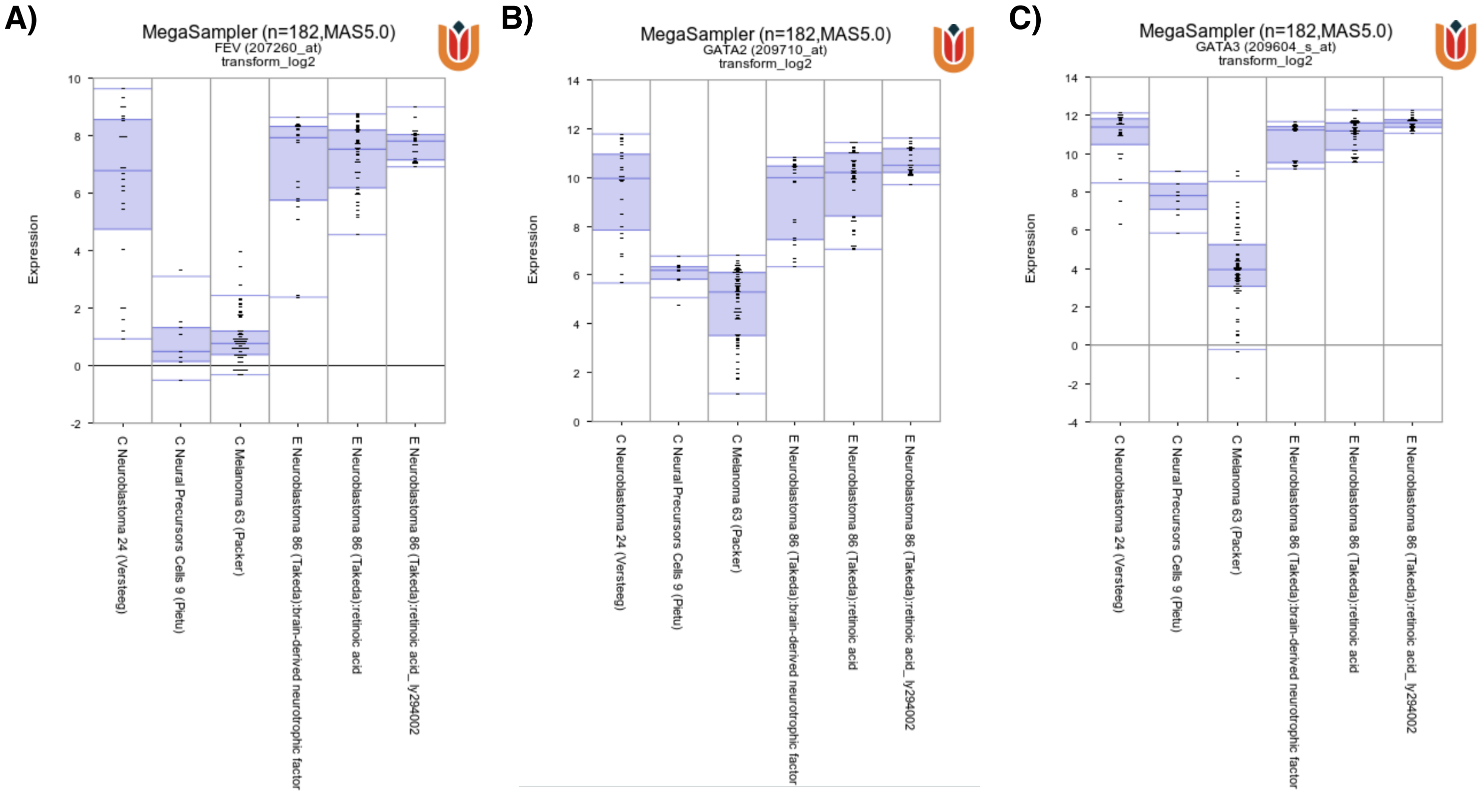
A)
MegaSampler (n=182,MAS5.0)
FEV (207260_at)
transform_log2
Expression
C Neuroblastoma 24 (Versteeg)
C Neural Precursors Cells 9 (Pietu)
C Melanoma 63 (Packer)
E Neuroblastoma 86 (Takeda):brain-derived neurotrophic factor
E Neuroblastoma 86 (Takeda):retinoic acid
E Neuroblastoma 86 (Takeda):retinoic acid_ ly294002
B)
MegaSampler (n=182,MAS5.0)
GATA2 (209710_at)
transform_log2
Expression
C)
MegaSampler (n=182,MAS5.0)
GATA3 (209604_s_at)
transform_log2
Expression
